# Green synthesis of selenium nanoparticles from *Cassia javanica* flowers extract and their medical and agricultural applications

**DOI:** 10.1038/s41598-024-77353-2

**Published:** 2024-11-05

**Authors:** Mohamed K.Y. Soliman, Mohamed Abdel-Aal Amin, Abdelatti Ibrahim Nowwar, Mahmoud H. Hendy, Salem S. Salem

**Affiliations:** Botany and Microbiology Department, Faculty of Science, Al-Azhar University, Nasr City, Cairo, 11884 Egypt

**Keywords:** Biosynthesis, SeNPs, Antimicrobial, Antibiofilm, Antioxidant activity, *Vicia faba*, Biotechnology, Biomaterials

## Abstract

Nanostructured materials are advantageous within numerous fields of medicine owing to their intriguing qualities, which include their size, reactive surface, bioactivity, potential for modification, and optical characteristics. *Cassia javanica* flower extract was used as a chelating agent in an environmentally friendly process to create SeNPs FTIR, XRD, and TEM, SAED were utilized to analyze and characterize the synthesized. The findings showed that the MIC of Se NPs against *B. subtilis* and *S. aureus* was 500 µg/ml. Conversely, the MIC for *P. aeruginosa*, *E. coli*, and *C. albicans* were 125, 250, and 62.5 µg/ml, respectively. Hence, SeNPs considerably reduced the activity; the inhibition peaked at 77.6% at 250 µg/ml to reach 49.04% at 7.8 µg/ml. Which showed the greatest suppression of MRSA biofilm formation without affecting bacterial growth. SeNPs showed an intriguing antioxidant capacity, achieving an IC_50_ of 53.34 µg/ml. This study looked how soaking seeds before sowing them with Se NPs at 50, 100, and 200 ppm affected the plants’ development in different parameters, as well as their yield of *Vicia faba* L. The growth conditions were effectively increased by soaking application of various quantities of Se NPs. The highest values of dry weight/pod (g), number of seeds/plant, weight of 100 seeds (g), and number of pods/plant were caused by high concentrations of Se NPs, by 28.43, 89.60, 18.20, and 94.11%, respectively.

## Introduction

Nanotechnology has the power to transform agriculture and have a significant impact on crop and food production^1–3^. The physicochemical properties of nanoparticles are superior to those of bulk materials characteristics at ideal concentrations^4–7^ . In recent years, the usage of nanoparticles (NPs) in industry and agriculture has increased^8–11^. The advancement of nanomedicine has the potential to completely transform how we identify and manage a wide range of illnesses, including cancer and bacterial infections resistant to antibiotics^12–19^. SeNPs are one type of nanoparticle that has received a lot of attention lately because of its possible medicinal uses^20^. SeNPs have been shown to be less hazardous as the inorganic and organic forms of selenium^21^. Additionally, more research is being done on their potent antibacterial and antioxidant properties^18^. Green approaches have recently been offered as a substitute for the usage of harmful reducing substances^22–26^. Metabolites, which are present in fungi, bacteria, and plants, constitute biologically active compounds of biological significance that must be added in order enable the environmentally friendly synthesis of SeNPs to occur. Because the microorganisms used for NPs synthesis no longer require cell upkeep, using plant extracts for NPs production is thought to be a more permanent and straightforward process^28–30^. Additionally, plant extracts function as stabilizing as well as decreasing agents which encourage the synthesis of NPs via the primary classes of secondary metabolites they contain, including terpenoids as well as favonoids^31–33^. However, certain characteristics, like the antioxidant qualities present in plant extracts, can be enhanced during the production of NPs^34,35^. However, there are some negative effects of nanomaterials that can be neglected^36^. Both humans and animals require the trace element selenium (Se). Enhancing antioxidant activity in plants, animals, and people is linked to selenium, as it is a structural component of various enzymes having physiologically antioxidant capabilities, such as thioredoxin reductase and glutathione peroxidases^37^. Selenium affects plants in two ways: with low amounts, it encourages development and proliferation, while at elevated levels, it has harmful effects. Certain plant species, including tomato (*Solanum lycopersicum*) as well as bell pepper (*Capsicum annuum*), have shown benefits from selenium, including enhanced plant height, growth, and dry weight of leaves and roots^38^. Although the effects of high nano-Se concentrations in soaking form on plant growth and development are currently unknown, the antistressor, growth-stimulating, and insecticidal qualities of nano-Se make its application very alluring^39^. The advantages of nano-selenium among other naturally occurring selenium sources derive from its small size, porosity, and bio-dispersion. SeNPs exhibit great potential in numerous vital metabolic and physiochemical functions, thereby enhancing the development of plants, Because SeNPs absorbed slowly but oxidized quickly to selenite (forming the organic forms SeCys and SeMet) inside the plant, they had stimulatory effects^43^. According to reports, SeNPs are unique compounds that are less hazardous than existing seleno-species and have greater antioxidant qualities because of their zero-oxidation state^41^. *Cassia javanica* belongs to the Leguminosae (Fabaceae) family and the sub-family Caesalpinioideae. Naturally occurring in Malaysia, Southern China and the Philippines. Several pharmacological characteristics of *C. javanica* include antibacterial, anti-tumor, antidiabetic, and antioxidant properties^42^. SeNPs also exhibit a variety of biological activities, such as antibacterial, antioxidant, and anticancer properties^43,44^. It has been demonstrated that SeNPs guard against infections brought on by harmful bacteria in humans such as *E. faecalis*,* B. cereus*,* L. monocytogenes*, *S. agalactaie*, and *S. aureus*^45^. Furthermore, bacterial pathogen biofilms are important in a number of diseases, and their properties may significantly increase the level of antibiotic resistance within microbial populations^46^. Even while the matrix of biofilm can operate as a barrier to mobility, antibacterial drugs and immune system functions may become limited^47^. This situation necessitates a prompt reaction in addition to a creative strategy for developing novel, safe, and effective antibacterial drugs using nanotechnology. The objective of this work is to use *Cassia javanica* flowers for the biosynthesis of SeNPs for the first time. The characterization of Se-NPs has been done using FTIR, XRD, TEM analysis. Lastly, studies on their antibacterial, antibiofilm, and antioxidant qualities have been conducted. Furthermore, evaluate the effects of elevated nano-Se concentrations on biometric features, faba bean development, yield, and the build-up of protein, phenol, and carbohydrates in response to treatments.

## Materials and methods

### Preparation of plant flowers extract

*Cassia javanica* tree cultivated in the garden of the Faculty of Science, Al-Azhar University. The healthy and disease free medicinal plant *Cassia javanica* flowers were collected during the month of November 2023, from the garden region of the Faculty of Science, Al-Azhar University, Egypt following established protocol, and permission was obtained. Further, the plant material was identified at Department of Botany and Microbiology, Al-Azhar University, Egypt. *Cassia javanica* fresh flowers were collected and cleaned with Milli-Q sterile water. The flowers of *Cassia javanica* were shade-dried after being cleaned of debris using sterile water from distillation. A 500 ml beaker containing 10 g of dried *cassia javanica* flowers and 500 ml of H_2_O was used to create the extract. The mixture was brought to a boil for fifteen min, or until the colorless aqueous solution became yellow. To remove the biological materials, the resulting mixture was centrifuged at 1500 rpm for 5 min after being cooled, then filtered using filter-paper. To prepare it for use in the next study, the extract was stored at 25 °C.

### Biosynthesis of Se NPs

As described in a prior publication, the synthesis of SeNPs was carried out^48^. Flowers from Cassia javanica was used to make extracts, and 20 ml of the extract was added to 180 ml of 10 mM Na_2_SeO_3_. At 40 °C to feed 40 min, the reaction was stirred magnetically at a speed of 1200 rpm. The reaction was carried out for a full day at room temp, continuous magnetic stirring, in a darkened room. Se-NPs are being collected and dried to carry out different studies.

### Se-NPs characterization

FT-IR (JASCO., FT/IR-6100) was used to analyze the functional groups found in the Se-NPs that were generated. Se-NPs were mixed by KBr and then tightly packed into discs. FT-IR spectra were obtained by scanning the disks at 400–4000 cm ^− 1^. However, the crystallo-graphic appearances of the resultant Se NPs were determined using XRD forms of Se-NPs equipped with an inversely related contrary about Ni-filter Cu-Kk energy and operating at a potential of 40 kV as well as an output current of 30 mA. The study examined the crystalline composition of Se NPs across a general 2 h range of 10^o^ to 80^o^ C. It was possible to determine the dimensions and form of the generated SeNPs by using TEM, JEOLـ2100 to watch their droplet encompassing process, and this involves laying a drop of NPcontaining the solution on wrapped carbon grids constructed from copper along with vacuum-desiccating this overnight.

### Antimicrobial activity

*Bacillus subtilis* (ATCCـ6633), *Staphylococcus aureus* (ATCCـ6538), *Pseudomonas aeruginosa* (ATCCـ9027), *Escherichia coli* (ATCCـ25922), and *Candida albicans* (ATCCـ10231) were among the many microbial strains used as specimens for analysis in the inquiry. Nutrient broth was used for growing a pure strain of microorganisms. The investigated microbes were evenly distributed using MullerـHinton agar on sterile petri dishes. A sterilized cork-borer was used to construct a well with a 6 mm diameter within each plate. To evaluate the antimicrobial effectiveness test, 100 ml of SeNPs were added to the well. After that, the plates were allowed to stand for 24 and 72 h, respectively, at 37 and 30 ºC and then the areas of inhibition were measured^49^. Utilizing the broth-based microdilution technique, the minimum inhibitory concentration (MIC) of SeNPs was investigated for several microbiological strains at doses that varied from 1000 µg ml^-1^ to 15.75 µg ml^-1^ . Initially sterilized MTP wells were filled with 100 µl of doubleـstrength MuellerـHinton (MH) broth. One hundred ml of the specimens in different concentrations were then added. A microbiological solution of cells was added to all but the negative control well. Positive control wells were filled with bacteria to see if MH broth would support microbial growth. The plates were then allowed to incubate for a full day at 37 °C. Utilizing a microplateـreader as well as the lowest dosage of the specimens that suppressed the microbes under test equivalent to positive as well as negative controls, one may determine the MIC in accordance with the Clinical and LaboratoryـStandards Institute (CLSI) guidelines^50^.

### Biofilm inhibition assay

Using MRSA, a clinically applicable strain with a powerful biofilm-forming agent, the MTP technique was used to determine the potential benefits of SeNPs for preventing or minimize the formation of bacterial biofilms. We altered the biofilm experiment from the earlier study in a number of ways^51^. In conclusion, varying dosages of Se-NPs were added to TSB Medias including MTP and supplemented with 1% glucose. The bacteria under examination were cultivated on MTP for 48 h at 37 °C after being diluted 1:100 in TSB. The planktonic cells were removed from the plates, and during the incubation phase, their growth density (OD: 620 nm) was measured. After removing every well material to prevent disturbing the biofilms that had grown, the biofilm was fixed for 10 min using 200µL of 95% methanol as the solvent. It was then washed 3 times using phosphateـbuffered saline (PBS) at a pH of 7.40. After adding 0.3% w/v crystal violet to the 200µL wells, they were left to remain at a room temperature approximately 15 min. Finally, the plates were washed using distilled water and then the wells were filled using a 30% acetic acid solution for the quantitative measurement of biofilm formation. The absorbance value was evaluated at O.D. 540 nm utilizing the STATFAX-USA microplate reader. The results were confirmed by comparing the comparative wells that were treated versus untreated^52^.

### Antioxidant activity

The capacity of biogenerated SeNPs (ranging from 1000 to 31.25 µg/ml) to scavenge DPPH radicals has been evaluated. A solution consisting of 1, 2-diphenyl-1-picrylhydrazyl (DPPH) radicals was produced utilizing 95% ethanol. Two hundred ml of Se-NPs concentration were well shaken with 800 µl of DPPH solution, and the combination was then allowed for 0.5 h at 25 °C in total darkness. Following that, centrifugation continued for a further five min at 13,000 rpm^53^. The wavelength of the absorbance has been determined at 517 nm for each concentration and compared to a blank. Ascorbic acid was used as a standard level. The previously described formula has been utilized to calculate the DPPH scavenging activity (%) for both standard and customized amounts of Se-NPs to evaluate their antioxidant capacity:

$$DPPH\,(\%)=((Aborb.of control -Aborb. Of sample )/ Aborb.of control) \times\,100$$

### A pot experiment was conducted to show the influence of a Se NPs soaking at different concentrations on Faba bean plants

The seeds of *Vicia faba* L. variety Nubaria 1. (faba bean) were sourced from Agriculture Ministry, Agricultural Research Centre, Giza, Egypt. Egypt. A pot experiment was conducted in botanical garden, Faculty of science, Al-azhar University, Egypt, utilizing sandy loam soil. For the experiment, treatments were categorized into four groups: 1- control, without treatments (soaking in fresh water), 2- seeds soaked in 50 ppm of Se NPS, 3- seeds soaked in 100 ppm of Se NPS, and 4- seeds soaked in 200 ppm of Se NPS. Plant samples were harvested at 45 after sowing, and assessed for morphological and biochemical characteristics. Morphological assessments included shoot and root lengths, fresh weights of shoot and root, and leaf count. Biochemical analyses encompassed chlorophyll (a & b), total chlorophyll, shoot phenol, carotenoids, shoot proline, shoot carbohydrate, and shoot protein. After 180 days, dry weight/pod, 100 seed weight, number of seeds/pod, number of pods/plant and levels of carbohydrates, protein, and phenolic compounds were evaluated on five hand-selected plants per treatment, providing insights into plant development, growth, and treatment efficacy. Controlled growth measurements and biochemical analyses were conducted to further comprehend the impact of these factors, elaborated upon in subsequent sections.

### Pigment and carotenoid identification

Fresh green leaves weighing one gram were processed according to the technique outlined by Vernon and Selly^54^.The quantities of chlorophylls (a and b), as well as their total amounts in plant tissues by Vernon and Selly^55^. For the estimation of carotenoid chemical composition, the method described by Lichtenthaler, et al.^56^.

### Extraction and quantification of soluble carbohydrates

Plant tissue was ground into a fine powder after being dried at 60 °C until a constant dry weight was achieved. A conical flask holding 100 ml was filled with one gram of the powder that would be examined. To this was added 5 ml of 2% phenol water and 10 ml of 30% trichloroacetic acid. After giving the mixture a good shake, it was left to sit for the entire night before filtering. Following that, the volume of the filtrate was lowered to 50 ml^56^. The anthrone technique for quantifying soluble carbohydrates^57^.

### Quantification of water-soluble proteins

The plant shoots were dried at 60 °C until a constant dry weight was reached to eliminate the water-soluble proteins. One gram of the finely ground, dried shoot powder was added to a cone. Next, 10 ml of distilled water were combined with 5 ml of 2% phenol water. Following filtration of the reaction mixture, the filtrate’s ultimate volume of 50 ml was adjusted using distilled water.

The contents of proteins were ascertained according to Lowry, et al.^58^.

### Estimation of total phenolic compounds

Folin-Ciocalteu reagent was used to detect phenols (mg 100 g\1 DW), as stated in^59^. One ml (1:1) of water: acetone solution was used to extract the 0.2 gram sample. The combination underwent a 30-second vortex. The tubes were spun for 10 min at 4 ℃ at 17,500 g. Five ml of distilled water, two ml of the Folin-Ciocalteu reagent, fifty ml of 20% Na_2_CO_3_, and fifty ml of the supernatant were put to a test tube and vortexed for thirty seconds. The samples spent thirty min in a water bath set at 45 ℃.   Ultimately, a plasticـcell in a UV-vis spectrophotometer was used to take the reading at an absorbance of 750 nm.

### Statistical analysis

Statistical evaluation was calculated using the Minitab 18 application. Tukey’s test (honestـsignificant difference) was used for post hoc evaluations, with a significance threshold of *p* < 0.05.

## Results and discussion

### Biosynthesis and characterization of Se nanoparticles

**Fig. 1:**
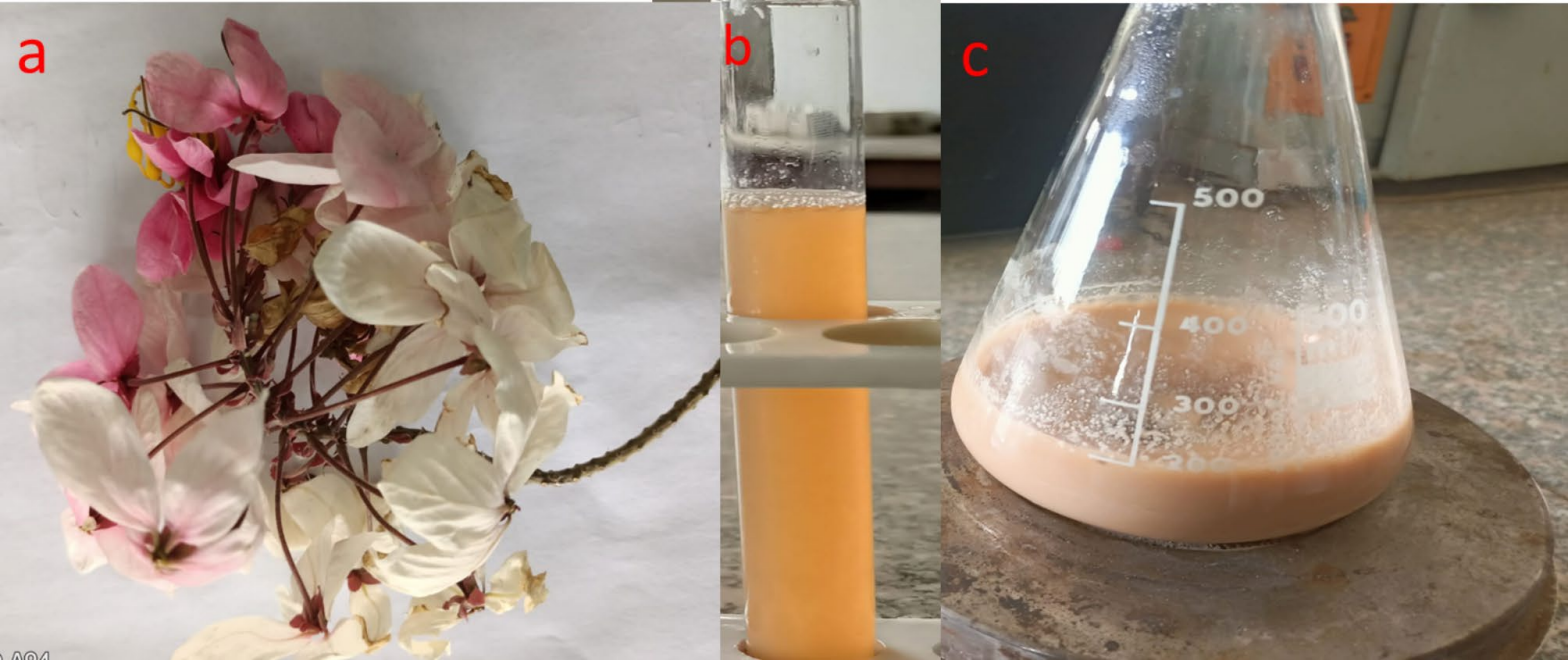
(**a**) *Cassia javanica* flowers, (**b**) plant extract, and (**c**) Biogenic Se NPs.

In this work, sodium selenite was converted to SeNPs by using a watery extract of *Cassia javanica* flowers as a reducing agent. Natural, non-toxic, reasonably priced, and environmentally acceptable ingredients are employed as reducing, capping, and stabilizing agents in green synthesis^60^. As a result, the produced nanoparticles have less of an adverse effect on the environment, are more biocompatible, and have more potential uses in a range of industries. The metabolites of *Cassia javanica* flowers releases function as a capping and reducing agent in environmentally friendly methods of nanoparticle formation. The solution’s hue changed to light orange, signifying the synthesis of SeNPs (Fig. 1).

**Fig. 2:**
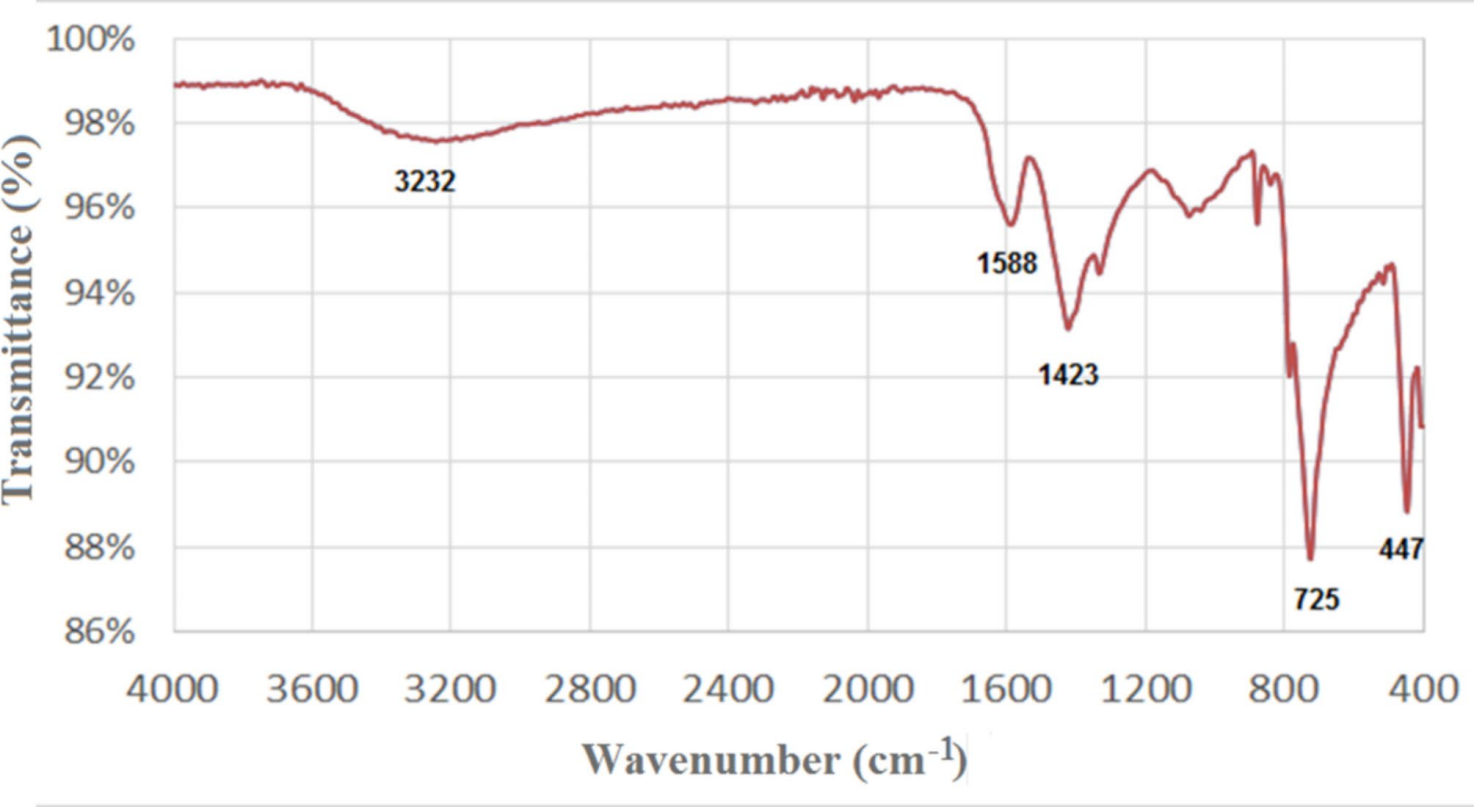
FTIR spectrum of SeNPs produced by *Cassia javanica* flowers.

FTIR analysis was conducted to confirm the presence of functional groups responsible for both the biogenic production and the long-term stability of Se NPs. The biomolecules found within Se NPs were documented within the 400–4000 cm^−1^ range. This observation validates the synthesis of Se nanocrystals. The distinct absorption bands of diverse functional groups distinctly demonstrate the adsorption of phytochemicals on the Se NPs’ surface^61^. Consequently, the phytochemicals present in plant extract serve a dual purpose, facilitating the formation of Se NPs by reducing selenium salt and stabilizing them through adsorption onto the NP’s surface. The purified SeNPs reduced by *Cassia javanica* flowers were analyzed by FTIR, revealing characteristic peaks at 3232 cm^−1^ for O-H stretching vibrations, 1588 cm^−1^ for tertiary amide, 1423 cm^−1^ for COO- group, and 725 cm^−1^ for C-H stretching vibrations (Fig. 2). In the end, peak 447 cm^−1^ indicates the presence of selenium in the nano-solution, which confirms its good formation from the plant extract. These comprehensive results indicate that the purified SeNPs sample has characteristic peaks of amide, carboxyl groups, etc.

**Fig. 3:**
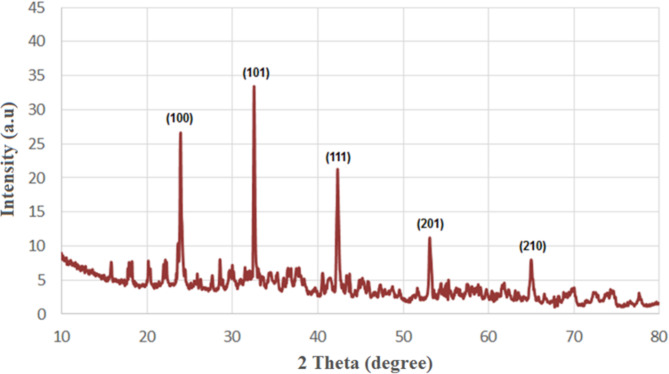
XRD pattern of SeNPs produced by *Cassia javanica* flowers.

XRD pattern has pointed sharpـpeaks, which validates that the synthesized SeNPs are crystalline in structure (Fig. 3). XRD validates the creation of Se NPs with crystalline structure^62^. The successful synthesis of Se NPs was confirmed by powder XRD. The well-crystalline nature of the produced SeNPs is demonstrated by the strong peaks in the diffraction pattern. The SeNPs produced through bio fabrication displayed a crystalline structure. The XRD pattern revealed 2θ values spanning from 10° to 80°. Several distinct peaks corresponding to SeNPs were observed at angles such as 100°, 101°, 111°, 201°, and 210°, and these angles matched the Miller indices.

**Fig. 4:**
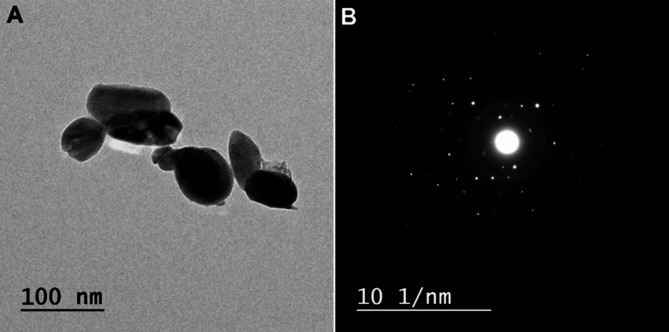
TEM image and SAED pattern of SeNPs produced by *Cassia javanica* flowers.

The purpose of employing TEM was to determine the specific size and shape of the SeNPs that were biosynthesized, as shown in Fig. 4A. This observation indicated the presence of spherical and irregular shapes with a narrow size distribution ranging from 35 to 100 nanometres. TEM was utilized to confirm the successful synthesis of Se using biological methods^60^. The primary goal of using TEM was to identify and characterize the precise morphology of SeNPs generated, as demonstrated before. The generated SeNPs’ SAED pattern indicated that they were crystal-like particles Fig. 4B. The diffraction ring represents the plane reflections of the crystallised selenium levels 100, 101,111, 201, and 210, which showed previous in XRD pattern.

### Antibacterial activity

The antimicrobial efficacy of Se NPs against unicellular fungal strains *C. albicans*, *B. subtilis*, *S. aureus*, *E. coli*, and *P. aeruginosa* was effectively assessed. The zone of inhibition of microbiological infections was ascertained by employing 1000 µg/ml of biosynthesized SeNPs. The biosynthesised SeNPs demonstrated strong antimicrobial action, with Bacillus subtilis showing the lowest zone of inhibition and Candida albicans showing the highest zone of inhibition (Table 1). We investigated the inhibitory effects of different concentrations of Se NPs (15.62–1000 µg/ml). The findings showed that the MIC of Se NPs against *B. subtilis* and *S. aureus* was 500 µg/ml. Conversely, the MIC for *P. aeruginosa*, *E. coli*, and *C. albicans* were 125 µg/ml, 250 µg/ml, and 62.50 µg/ml respectively (Table 1). The Agar diffusion test has a correlation with the MIC values. The antibacterial efficacy of those NPs made from plant extracts may be due to their existence of phytochemicals including flavonoids, terpenoids, alkaloids, as well as additional bioactive components^63^. These plant-based chemicals could block the enzymes needed for microbe viability that replicate DNA and expand genes. Moreover, these substances cause pathogenic cell death by switching on the permeability of the cell walls along with cell membrane^64^. Another reason why SeNPs could be antibacterial is that they might cause microbial cells to die by inactivating enzymes or producing oxygen species that are reactive^65,66^. SeNPs were discovered to have dose-dependent action toward all evaluated bacterial strains when their effectiveness as antibacterial agents was investigated against a variety of gram-positive ( *S. aureus* and *Streptococcus epidermidis*) along with gram-negative (*P.aeruginosa* and *E. coli*)^67^. SeNPs bio-synthesized via leaf extract of Mountain persimmon exhibited antibacterial action toward *S. aureus* and *E. coli*^68^. Gram-positive bacteria typically have a greater cell wall made mostly of peptidoglycans, whereas Gram-negative bacteria possess an exterior cell membrane made of lipopolysaccharides and an inner cell wall with a thin coating of peptidoglycans^69,70^. Gram-positive bacteria as well as Gram-negative bacteria have negatively charged cell walls, although the cell walls of Gram-negative bacteria are often more negatively charged^70–72^.

**Table 1:** Antimicrobial activity of SeNPs.

Microbial strains	IZ (mm)	MIC(µg/mL)
*S. aureus*	14.1 ± 0.5c d	500
*B. subtilis*	13.2 ± 0.21 d	500
*E. coli*	14.7 ± 0.55 c	250
*P. aeruginosa*	15.9 ± 0.28 b	125
*C. albicans*	18.2 ± 0.3 a	62.5
*HSD at 0.05*	1.006	-

### Antibiofilm

**Fig. 5:**
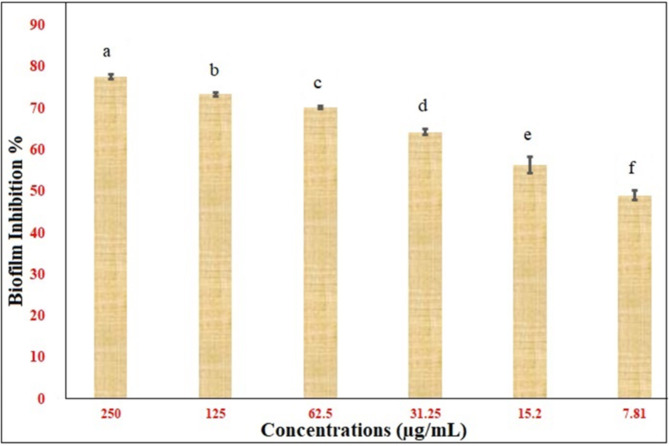
Antibiofilm assay of biosynthesized Se-NPs.

In the current study, the antibiofilm effect of SeNPs demonstrated a variety of results against MRSA. Hence, SeNPs considerably reduced the activity; the inhibition peaked at 77.6% at 250 µg/ml to reach 49.04% at 7.8 µg/ml. which showed the greatest suppression of MRSA biofilm formation without affecting bacterial growth (Fig. 5). SeNPs can be applied to the outer layer of medical equipment to stop the production of biofilm. Compared to unprotected polycarbonate areas, they significantly slowed the development of *S. aureus* on the outside by 91% and 73% after a period of 24 h and 72 h, respectively^73^. Specifically, at an amount of 16 mg/L, SeNPs generated by *Bacillus* sp. MSh-1 inhibited the development of biofilms by *S. aureus*, *P. aeruginosa*, and *P. mirabilis*. The reduction in biofilm development was 65.7% for *P. aeruginosa*, 58% for *S. aureus*, and 46.6% for *P. mirabilis*^74^. Based on the outcomes of their antibiofilm activity, Se-NPs might be considered a potential treatment option for inhibiting the formation of biofilms. Biofilms formed from MRSA have been extensively studied for this inhibition; significant reduction was noted, with a reduction in biofilm formation of over 50%. These results are in line with those of another researcher who found all of the tested strains showed substantial inhibition (*p* < 0.01; *p* < 0.001) at a dose of 6.4 µg/ml, resulting in a more than 50% reduction in production^75^. SeNPs may be helpful weapons in this struggle over the formation of biofilms. The antibiofilm effect of SeNPs, whose are produced by *P. vermicola* and consist of a median size of particles of 28 nm, prevents harmful bacteria like S. aureus as well as others from forming biofilms 95% of the time^76^.

### Antioxidant activity

Cell death is usually caused by reactive oxygen species (ROS), which are produced by biological activities and oxidatively degrade the biological elements^77^.

**Fig. 6:**
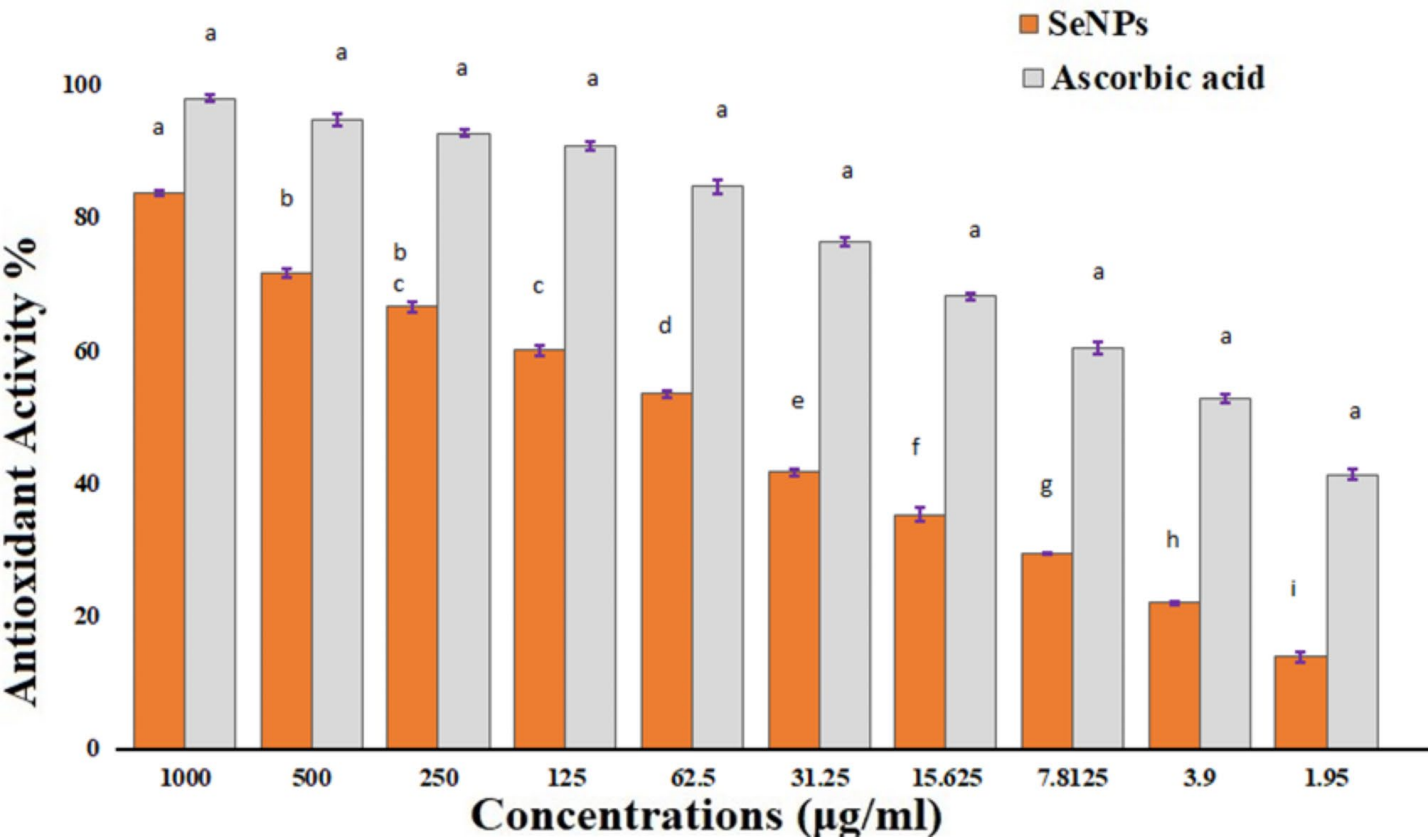
Antioxidant assay of Phyto fabricated Se-NPs.

Following that, antioxidant chemicals were used to reduce the negative impacts of ROS. Moreover, antioxidants are widely employed as medical treatments due to their anti-atherosclerotic, anti-mutagenic, anti-inflammatory, cancer prevention, and antimicrobial effects^78,79^. The antioxidant activity of these produced SeNPs was assessed in the present investigation using the DPPH freeـradical assay. Figure 6 illustrates the antioxidant capacity of Se NPs at different dosages using ascorbic acid provides a positive control. The results showed that the IC_50_ of Se-NPs was 53.34 µg/L (Fig. 6). Further studies evaluated the antioxidant activity of Se NPs made with different techniques, and these studies showed that NPs have excellent antioxidant capabilities^80^. Selenium probably correlates towards the strong antioxidant properties of SeNPs to be it is necessary for increasing the effectiveness of selenium-containing enzymes such as glutathione peroxidase helps protect tissues and cells from radical destruction^80^. Kokila et al.., have demonstrated that phyto-synthesised Se NPs with a diameter of 16 nm had a value for the EC50 of 22.5 µg/ml^81^. Qiu et al.., produced pectin decorated SeNPs measuring 41 nm and determined that their EC50 value was 500 µg/ml in a different publication^81^.

Thus, phyto synthesis SeNPs have an opportunity to serve as a natural antioxidant incorporating ingredient in food packaging components and to replace artificial antioxidants due to their excellent biocompatibility^82,83^. Additionally, it is shown that the SeNPs have an extensive amount of potential for scavenging dangerous free radicals, demonstrating significant antioxidant potential^84^. Abduljabbara et al., demonstrated the antioxidant capacity via using the DPPH test, the antioxidant capacity IC_50_ of E. retusa extract was 0.054 mg/ml while SeNPs was 0.247 mg/ml. The results for the metal nanoparticle solutions shown noticeably less potency than the plant extract, which demonstrated far higher efficacy^85^.

### Growth traits

SeNPs exhibit great potential in numerous vital metabolic and physiochemical functions, thereby enhancing the development of plants, the use of SeNPs boosted tobacco, tomato, and mustard plant growth^86^. Results in Table 2. showed that, soaking of SeNPs at 50,100 and 200 ppm caused significant increase in shoot and root height, fresh weight of shoot and root and number leaves of faba bean plants. Se NPs plays a crucial role in promoting plant growth through improving glucose metabolism, restoring the ultrastructure of chloroplasts, speeding up the synthesis of chlorophyll, and halting the breakdown of chlorophyll^87^. Also, Se-NP significantly increased growth traits and the overall leaf area cm^2^/plant, notably at 6.25 M concentration, which Se NPs caused elevated levels of the growth indole acetic acid, gibberellic acid, and cytokines in treated plants^88^. In other research SeNPs changed the photosynthetic pigments, total flavonoids, phenol content, and total soluble sugars, in the plants, all of which had an effect on the growth of groundnut cultivars^89^. SeNP doses range 50 and 100 mg kg^-1^ were shown to considerably improve root system growth (> 40%) and organogenesis in plant tissue culture^90^. Additionally, SeNPs support organogenesis and root formation. It has been demonstrated that trace amounts of Se promote development in potato, lettuce, ryegrass, and Brassica oleracea plants^91^.

**Table 2:** Growth traits in response to different concentrations of phytosynthesized SeNPs.

Growth Traits					
Treatments		Shoot lengths (cm)	Root lengths (cm)	Fresh weight of shoot	Fresh weight of root
Control	0 ppm	46.76 ± 2.01b	6.12 ± 1.04b	18.05 ± 0.25c	3.25 ± 0.43c
Se NPs	50 ppm	51.33 ± 3.21a	8.67 ± 0.92a	21.31 ± 2.24b	4.2 ± 0.48b
	100 ppm	56.67 ± 2.305a	8.9.08 ± 1.05a	23.17 ± 3.65a	5.17 ± 0.19a
	200 ppm	58.77 ± 3.136a	9.21 ± 0.84a	24.03 ± 1.87a	5.76 ± 0.60a
HSD at 0.05		8.25	1.32	2.35	0.73

### Pigments contents

**Fig. 7:**
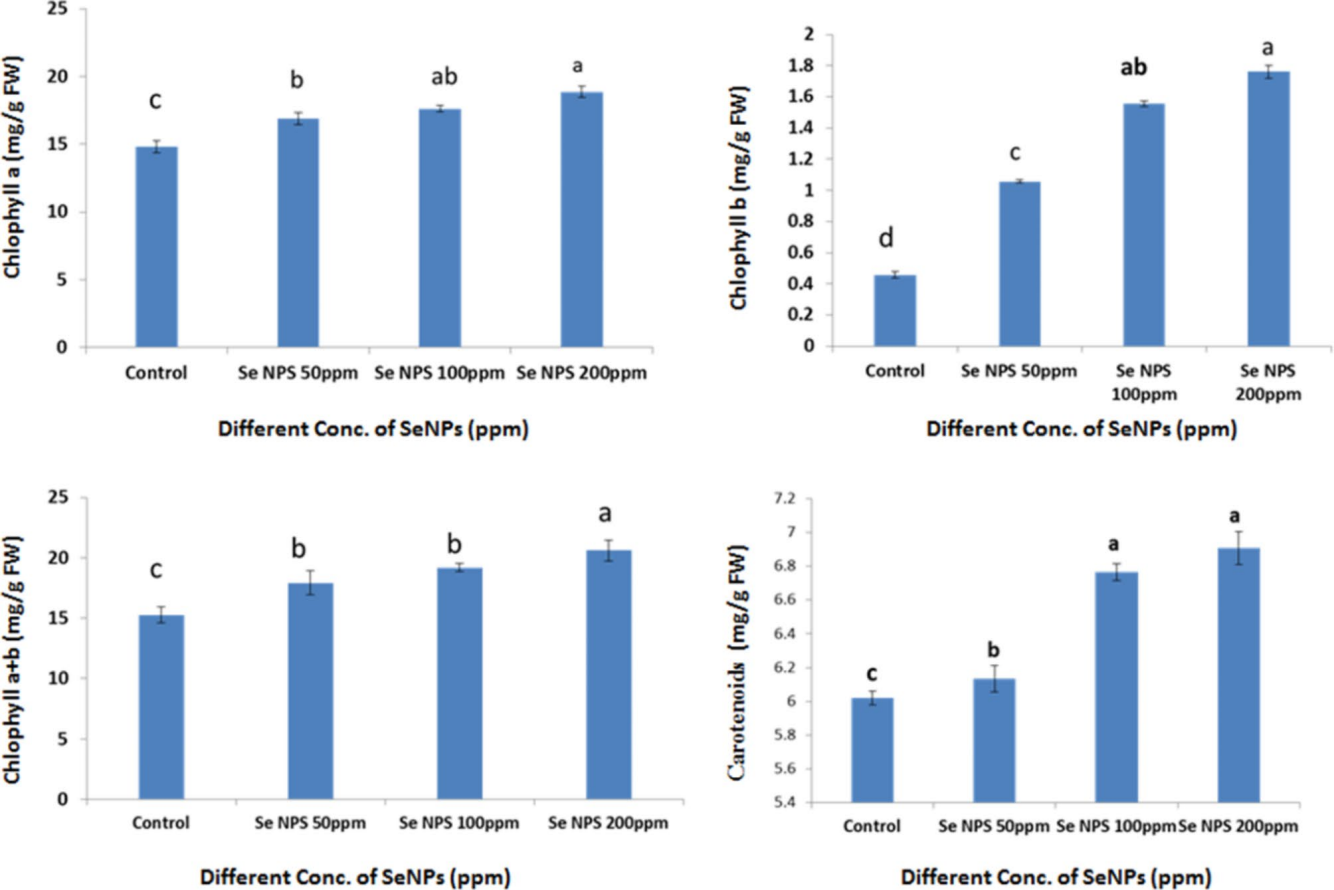
Effect of Se NPs at different concentration on pigments of Faba bean leaves.

Data in Fig. 7, appeared that Se NPS at 50, 100 and 200 ppm improve contents of chlorophyll a, b,a + b and carotenoids. This is may be as a reason of plants that are exposed to nanoparticles has a considerable increase in chlorophyll, which enables them to generate more complexes for light harvesting, which enhances the absorption of light energy^92^. Also, SeNPs could scavenge ROS and shield chlorophyll from oxidative damage, maintaining the content of the molecule. Plant biological systems depend on photosynthetic pigments as key sources of energy. These pigments are an important indicator of photosynthesis, and any changes have a concomitant impact on metabolism. In another study note that, bio-SeNPs dramatically raised the chlorophyll contents when compared to control^93^. The maximum value of chlorophyll contents was observed at 200ppm, which raised the doses of total chlorophyll, chlorophyll a, and chlorophyll b by 21.83, 24.85, and 24.04% (bioSeNPs), which can be attributed to the increased concentration of Se that protects photosynthetic pigments. An increased capability for antioxidant enzymatic activity caused by bio-SeNPs may be linked to a higher chlorophyll concentration^94^. Bio-SeNPs promote cellular defense against ROS accumulation and enhance osmotic substances by maintaining osmotic potential throughout the cytoplasm and vacuoles during metabolic processes. Generally speaking, the metallic nanoparticles (NPs) can enhance the absorption of light through chloroplasts by elevating the osmolyte levels and The response of expression of gene to light picking complex II^95^. Application of SeNPS had an impact on the amount of chlorophyll, particularly at higher concentrations of 150 µmol L^−1^).

### Soluble carbohydrates, proteins and phenol contents

**Fig. 8:**
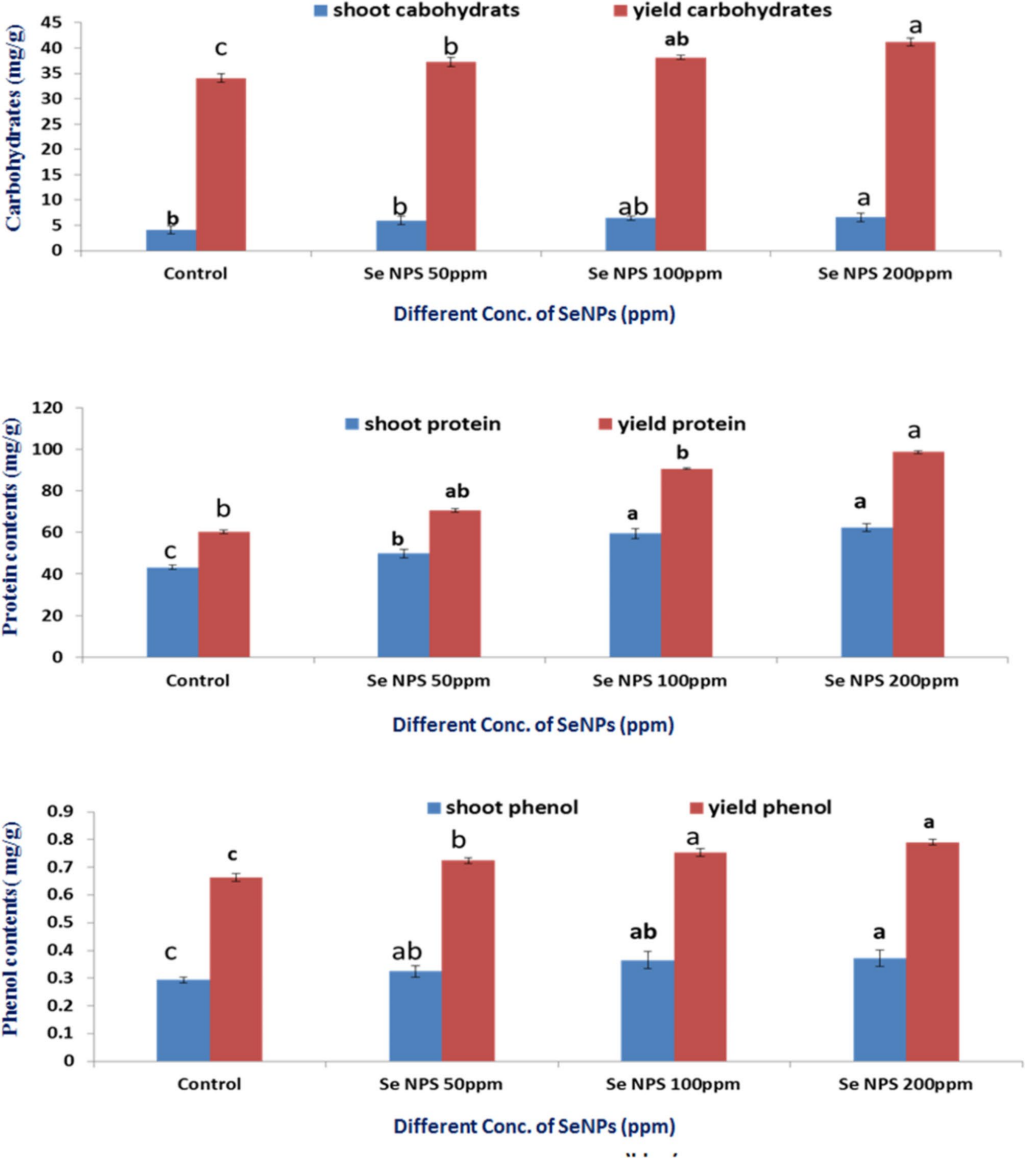
Effect of Se NPs at different concentration on carbohydrates, protein and phenol contents of Faba bean shoot and yield.

Our investigation demonstrated that the total soluble carbohydrates, protein and phenol contents of shoot and yield appeared increased trend with increasing SeNPs concentrations from 50 ppm up to 200 ppm (Fig. 8). In agreement with our study the authors^88^ which found that the biochemical measurements of the carbohydrates, protein and phenol contents in leaves of cowpea plants were improved by the application of Se-NP, especially at 6.25µM concentration. In comparison to their untreatedـcontrol plants, foliar use of various doses of nano-Se up to 10 µM was successful in boosting the amounts of total carbohydrates, and crude proteins in leaves. In our study Se NPs at 200 ppm appeared the highest values of carbohydrate’s, protein and phenol by 59.71, 44.17 and 26.60% respectively in shoot and by 20.96, 63.63 and 16.96% respectively in yield^96^. In another study on celery plant, the use of 5 mg/L of Se NPs as a foliar treatment increased the total phenols of leaves by (21.4%) compared to control^97^.

In the same concept, the researchers showed that foliar application of nano-Se up to 10 µM proved beneficial in enhancing yield (measured as 100 seed weight) In the same concept, the researchers showed that foliar application of nano-Se up to 10 µM proved beneficial in enhancing yield (measured as 100 seed weight).

### Yield characters

Data in Table 3 appeared that Se NPs in soaking form at different concentrations caused significant increment of yield parameters (dry Weight / pod (g), number of seeds/plant, weight of 100 seeds (g) and number of pods/plant). Se NPS at high concentration caused the highest value of dry Weight / pod (g), number of seeds/plant, weight of 100 seeds (g) and number of pods/plant by 28.43, 89.60, 18.20 and 94.11% respectively. In the same concept, the researchers showed that foliar application of nano Se up to 10 µM proved beneficial in enhancing yield (measured as 100seed weight)^96^. The use of SeNPs are among the most crucial elements in raising crop yields and agricultural production^98^. In a pot experiment on cluster bean plants, observed that Se NPS at 400 mg had higher yield, chlorophyll (a, b), free amino acids, total chlorophyll, leaf nitrate, anthocyanin, carotenoids, L-proline, and protein^99^. Additionally, nano-Se at 1.27 mM as a foliar application had a growth-stimulating impact that increased pod number, yield, and seed weight^100^.

**Table 3:** Yield traits under different concentration of biogenic Se NPs.

Yield Traits					
Treatments		Dry Weight / pod (g)	No. of seeds/plant	Weight of 100 seeds (g)	No. of pods/plant
Control	0 ppm	4.22 ± 0.083c	16.35 ± 0.44d	104.25 ± 0.25b	4.25 ± 0.43c
Se NPs	50 ppm	4.66 ± 0.089b	20.8 ± 0.42c	122.21 ± 1.24a	5.5 ± 0.45b
	100 ppm	4.81 ± 1.305b	28.9 ± 1.25b	120.27 ± 2.65a	7.75 ± 0.34a
	200 ppm	5.42 ± 1.136a	31 ± 0.74a	123.23 ± 1.87a	8.25 ± 0.32a
HSD at 0.05		0.30	2.42	5.35	0.79

## Conclusion

A safe and effective procedure for synthesizing Se NPs without harming the environment is provided by *Cassia javanica* flower extract. These biosynthesized Se-NPs were characterized using FTIR, XRD, and TEM, and SAED analysis. SeNPs were have antimicrobial potency versus *C. albicans*,* B. subtilis*,* S. aure*us, *E. coli*, and *P. aeruginosa*. Se NPs was shown an antibiofim activity versus biofilm producer *S. aure*us (MRSA), Additionally, SeNPs’ antibacterial and antibiofilm properties depend on their concentration. They also exhibit antioxidant properties and the ability to scavenge free radicals. In addition to significant variations in faba beans when different doses of nano-Se are supplied. The plants with the highest accumulation of protein, carbs, phenol, and pigment contents treated with nano-Se demonstrated the highest tolerance to the material. Se NPs demonstrated the highest production, biofortification levels, seed weight and quality, and resistance to elevated Se values among the four treatments that were examined. The characteristics of the Se biofortification of *Vicia faba* plants that have been discovered offer excellent chances for the development of functional foods using Se NPs.

## Data Availability

The datasets generated during and/or analyzed during the current study are available from the corresponding author on reasonable request.
